# Cool Solutions in Hot Times: The Case for Digital Health in Heatwave Action Plans

**DOI:** 10.2196/66361

**Published:** 2025-09-05

**Authors:** Maria Daniel Loureiro, Neil Jennings, Emma Lawrance, Daniela Ferreira-Santos, Ana Luísa Neves

**Affiliations:** 1 Faculty of Medicine, Universidade do Porto, Porto, Portugal; 2 Grantham Institute - Climate Change and the Environment, Imperial College London, London, United Kingdom; 3 Climate Cares Centre, Institute of Global Health Innovation, Imperial College London, London, United Kingdom; 4 Institute for Systems and Computer Engineering, Technology and Science, Porto, Portugal; 5 CINTESIS@RISE, Centre for Health Technology and Services Research, Faculty of Medicine, University of Porto, Rua Dr. Plácido da Costa, Porto, 4200-450, Portugal, 351 220 426 566; 6 Department of Primary Care and Public Health, Imperial College London, London, United Kingdom

**Keywords:** climate change, digital health, telemedicine, disaster planning, health information systems

## Abstract

This viewpoint highlights the critical need for proactive and strategic integration of digital health tools into heat-health action plans (HHAPs) across Europe. Drawing insights from the digital health surge during the COVID-19 pandemic and recent heat-related health impacts, we identify response gaps and suggest specific strategies to strengthen current plans. Key recommendations include leveraging mobile health communication, expanding telemedicine usage, adopting wearable health monitoring devices, and using advanced data analytics to improve responsiveness and equity. This perspective aims to guide policymakers, health authorities, and health care providers in systematically enhancing heat-health preparedness through digital health innovation.

## Introduction

Europe’s three warmest years ever recorded have all occurred since 2020, with 2024 setting a new record as the warmest [1]. In 2024, Europe experienced an average temperature of 10.7°C, exceeding 1.5°C above the pre-industrial level—a threshold set by the Paris Agreement to significantly reduce the risks and impacts of climate change. Globally, heat stress reached unprecedented severity, with approximately 44% of the Earth’s surface experiencing strong to ’extreme heat stress,’ a significant increase over the historical maximum [2].

The escalating frequency and severity of heatwaves (periods of abnormally hot weather lasting from 2 days to months [3]) impose a major physiological strain on individuals, potentially resulting in acute illnesses such as heat exhaustion and heatstroke. Heatwaves also exacerbate the risks associated with non-communicable diseases such as respiratory diseases, mental health issues, cardiovascular and renal diseases, or diabetes [4,5]. The impacts of extreme heat are disproportionately felt by vulnerable populations, such as the elderly and individuals with pre-existing health conditions. Additionally, these severe temperature episodes substantially strain health care systems worldwide, increasing demand on emergency services, hospital admissions, and ambulance callouts [6-8].

Heat-health action plans (HHAPs) are structured, multisectoral frameworks [9] that outline preventive and responsive measures to reduce the health impacts of extreme heat events. In accordance with guidance from the WHO Regional Office for Europe, they should include aspects such as early warning systems, public information plans, health care preparedness measures, and real-time surveillance of heat-related health outcomes [10]. Their objective is to minimize heat-related morbidity and mortality, particularly among high-risk groups.

Despite the clear necessity and well-documented effectiveness of such plans, significant gaps remain in their implementation across Europe. According to recent assessments by the European Environment Agency, only about half—21 out of 38—of European countries currently have operational HHAPs [11]. Furthermore, significant variability exists in the scope, responsiveness, and implementation of existing HHAPs, with many plans lacking essential components such as real-time health surveillance, targeted risk communication, or adequate integration with health care services. This heterogeneity has left large segments of the population insufficiently protected during extreme heat events. The limited effectiveness of these plans was evident during the summer of 2022, when, despite the activation of HHAPs across several European countries, more than 60,000 heat-related deaths were reported—a figure that underscores the partial and uneven success of current strategies [12]. Addressing these deficiencies by strengthening the adoption and quality of HHAPs is therefore not only urgent but ethically imperative to protect populations increasingly vulnerable to climate extremes.

To address this gap, this viewpoint aims to guide policymakers, health authorities, and health care providers in systematically enhancing heat-health preparedness through digital health innovation. Drawing from the digital health advancements that took place during the COVID-19 pandemic, we propose specific strategies for integrating targeted digital communication, telemedicine, and health data modeling into future HHAPs.

## Digital Innovation in Heat-Health Preparedness: Insights From the COVID-19 Response

The COVID-19 pandemic highlighted a powerful example of how rapidly health care systems can innovate, particularly in terms of digital health tools [13]. The swift implementation of digital technologies across health systems, from mobile health apps and telemedicine to real-time data dashboards for disease monitoring, transformed public health management during the pandemic [14]. These tools were instrumental in improving patient surveillance, ensuring continuity of care, and supporting effective communication strategies between health authorities and the general population [15].

The pandemic experience also revealed key operational needs that digital interventions addressed effectively, which are also directly applicable to heat-health preparedness: the necessity of establishing direct channels of communication with the public, ensuring continuous access to health care services during crises, and developing the capacity to accurately model risks and adapt response strategies in real time.

To operationalize these lessons, a range of digital health strategies can be mobilized to strengthen existing plans and address current gaps in heatwave preparedness.

## Rethinking Public Heat Communication Through Digital Channels

Effective communication is essential for raising awareness about the health impacts of heatwaves and promoting protective behaviors among the general public, vulnerable groups, caregivers, and organizations. Digital strategies can play a key role in sharing essential information such as identifying at-risk individuals (eg, those on psychoactive medications [16]), recognizing warning signs of heat-related illness, and locating available cooling centers. However, many countries continue to limit this information to institutional websites, which may not be easily accessible or sufficiently engaging to the broader population.

A more effective strategy would involve leveraging the digital infrastructure established during the COVID-19 pandemic to disseminate information [17,18]. National health authorities can repurpose mobile applications and social media channels to deliver critical information and issue public health guidance more directly and broadly. For example, during the pandemic, mobile applications were widely used to provide behavioral recommendations and assist with contact tracing [19]. Notably, the NHS COVID-19 app prevented approximately 1 million cases, 44,000 hospitalizations, and 9600 deaths during its first year of operation in England and Wales [20]. Similarly, social media platforms (such as Twitter) were used successfully by experts to disseminate public health messages at scale [21]. These tools could be readily adapted for heat-health communication. In addition, integrating messaging into health apps that are already in use by at-risk groups, such as for diabetes management or mental health platforms, could enable targeted outreach and ensure relevant information reaches those most in need [22,23]. Mobile health apps with monitoring functions, including those that track physiological data or geolocation, could be used to deliver personalized heat warnings based on user-specific risk profiles. Recent reviews have highlighted how such functionalities can enhance health communication and enable more proactive interventions during climate-related events [24]. The utilization of existing digital tools would not only leverage the substantial investments made in developing these apps but also enhance the responsiveness of health systems communication strategies to extreme weather events.

## Telemedicine as a Climate Adaptation Tool

The use of telehealth services could play a vital role in maintaining essential care during heatwaves, a time when populations are advised to limit outdoor activities. By enabling remote consultations, telemedicine reduces exposure to heat during travel or in-person visits. Health professionals acknowledge that implementing telemedicine during heatwaves, particularly for patients with chronic conditions or reduced mobility, could significantly enhance clinical management through primary care [25].

In addition to improving access to care, virtual consultations have also been identified as a means to advance environmentally sustainable health care delivery [26]. Virtual care reduces unnecessary patient transport, limits physical infrastructure needs, and minimizes the carbon footprint of routine care activities. This dual benefit of improving both health outcomes and environmental sustainability strengthens the case for mainstreaming telemedicine in the context of climate-related health risks.

Despite its promise, telemedicine remains largely absent from most heat-health action plans in Europe, even in areas where telehealth infrastructure was rapidly developed during the COVID-19 pandemic [27]. This gap reflects a missed opportunity to leverage proven tools in the face of increasing climate threats.

Moreover, telemedicine can play a critical role in pre-hospital triage, helping reduce pressure on overburdened emergency services during periods of high demand. For instance, during the COVID-19 pandemic, a structured multilevel telehealth service implemented in a resource-limited region in Brazil led to a 35% reduction in hospital admissions and a 40% decrease in mortality rates [28]. In contrast, during the 2011 heatwave, telecardiology support was successfully employed in Italy’s Apulia region to evaluate patients with suspected acute heart disease, improving care while minimizing unnecessary hospital admissions [29]. Incorporating such strategies into HHAPs would improve health care system responsiveness during heatwaves and help ensure continuity of care for at-risk populations. Clear operational guidance on activating telehealth during extreme heat should be a core component of future planning.

## Data Modeling for Adaptive Heat Responses

Heat action plans also should be capable of adapting to the dynamic interplay between health and heat. Integrating health data modeling and simulation into both the preparedness and response to phases can enhance the flexibility and precision of these plans [30]. To adequately capture the increased risk to vulnerable groups, activation thresholds for HAAPs should not only rely on ambient temperature, but also consider other meteorological parameters—such as humidity, nighttime temperatures, and heatwave duration—adjusted to the demographic and health profiles of the affected populations [31,32]. These may include factors such as age distribution, prevalence of chronic diseases, and proximity to health care services.

Combining health data with meteorological inputs also supports more informed policy making. This integration is essential for simulation and impact assessment studies, which can clarify the bidirectional relationship between causes and adaptations for extreme heat events. For instance, assessing the health benefits of air conditioning versus passive cooling methods in health care facilities requires balancing clinical effectiveness with environmental costs, energy use, and feasibility.

During heatwaves, near real-time indicators, such as emergency call volumes or hospital visits, paired with meteorological forecasts, can guide dynamic decision-making and allow for timely adjustments to response measures [33]. In France, the Heat-Health Watch Warning System is one example of such an integrated approach. It monitors both biometeorological indicators (which assess the impact of atmospheric conditions on living organisms) and health indicators such as mortality, emergency calls, and hospital visits for all causes and heat-related conditions. This comprehensive monitoring allows authorities, including civil protection and hospitals, to calibrate their response efforts [34]. Notably, during a severe heatwave in July 2006, the Heat-Health Watch Warning System saved more than 4300 lives in 18 days [33].

## Integrating Digital Innovation Into Heat-Health Action Plans Is a Public Health Imperative

Despite the success of such systems, digital tools remain underutilized in most European HHAPs. Only a few countries incorporate mobile apps or digital platforms into their plans, mainly for information dissemination. In fact, most current strategies still rely on more traditional channels such as telephone hotlines (eg, in Germany, Belgium, Portugal, and Romania) or weather-warning websites (eg, in Slovenia and Italy) [35]. While institutional websites, radio, television, and, more recently, social media continue to play a role [9], they are limited in their ability to deliver tailored messages to high-risk groups or to support interactive, two-way communication. The lack of integration with digital health infrastructures leaves many HHAPs poorly equipped to respond to the demands of increasingly frequent and severe heat events.

To enhance health system preparedness for extreme heat events, there is an urgent need for forward-looking, adaptable strategies that harness the full potential of digital technologies. These not only enable faster and more targeted interventions but also support more anticipatory and scalable public health responses. Realizing this potential requires more than the deployment of new technologies; it demands a shift in public health strategy. A systems-thinking approach is essential, one that embeds digital innovation into the core of heat-health planning and ensures coordination across sectors and levels of care. Such an approach supports both rapid emergency response and long-term resilience in the face of escalating climate risks.

It is, however, imperative to critically assess the environmental impact of these technologies. While telemedicine and digital consultations can significantly reduce carbon emissions by minimizing patient travel [36], the broader digital infrastructure also contributes to greenhouse gas emissions. Data centers, which power digital health services, account for approximately 1% of global electricity consumption [37]. A comprehensive evaluation of the environmental footprint of digital health interventions is therefore essential.

To the same degree, the accessibility of digital health policies should be assessed. While these interventions may not reach everyone equally—particularly older adults or individuals with limited digital literacy [38]—they can free up capacity in the system. The broad implementation of digital solutions can improve accessibility by allowing more focused and personalized outreach to those who may benefit from direct or non-digital support. These tools may also reduce geographic inequalities by improving access to care for populations in remote or underserved areas. Interventions such as wearables for continuous vital signs monitoring or assisted teleconsultation do not require active technology use by the individual and can still be deployed effectively among these groups. Recent reviews have highlighted the growing role of wearable devices to monitor the health effects of heat events and support public health surveillance at scale [24].

Still, barriers such as language differences, limited access to digital devices, and unfamiliarity with technology remain significant challenges that may undermine the effectiveness of these interventions. Engaging community stakeholders in the design and deployment of digital health solutions can help ensure these interventions are culturally sensitive, user-friendly, and responsive to diverse population needs.

Integrating digital innovation into heat-health action plans is no longer optional; it is a public health necessity. As climate change accelerates the frequency and severity of extreme heat events, health systems must evolve accordingly. This viewpoint suggests that integrating digital tools into HHAPs is not simply an opportunity but an essential step toward modernizing public health preparedness in the face of climate change. We call on public health decision-makers to act swiftly and collaboratively to embed digital innovation within all stages of extreme heat response.

## Abbreviations

HHAP: Heat-health action plan
